# The role of soluble CD80 in patients with soft tissue tumors

**DOI:** 10.1186/s13018-022-03283-2

**Published:** 2022-09-05

**Authors:** Yumi Matsuyama, Kunihiro Asanuma, Keisuke Yoshida, Tomohito Hagi, Takahiro Iino, Tomoki Nakamura, Akihiro Sudo

**Affiliations:** Department of Orthopaedic Surgery, Mie University Graduate School of Medicine, 2-174 Edobashi, Tsu City, Mie 514-8507 Japan

**Keywords:** Immune checkpoint protein (ICP), Soluble CD80, Prognosis

## Abstract

**Background:**

Immune checkpoint protein (ICP), which is a central factor group of the immune system, has been reported to have a correlation between the degree of its expression and the prognosis of patients with malignant tumors, and many inhibitors have appeared as therapeutic targets. On the other hand, a soluble form of ICP in circulating blood induced systemic immunosuppression. In this study, we investigated the relationship between the soluble form of CD80 (sCD80) which is a ligand for the inhibitory system CTLA-4, in blood, and clinicopathological parameters in patients with soft tissue tumors.

**Methods:**

A total of 119 patients with primary soft tissue tumors were enrolled in this study. The sCD80 levels were measured by enzyme immunoassay.

**Results:**

There were no significant differences in sCD80 levels between benign (34) and soft tissue sarcoma (STS) patients (85). In STS, the high-sCD80 group had significantly lower metastasis-free survival (MS) and lower overall survival (OS) than the low-sCD80 group at 5 years using the log-rank test (OS: high > 404 pg/mL, low ≤ 404 pg/mL, MS: high > 531 pg/ml, low ≤ 531 pg/ml). On multivariate Cox proportional hazard analysis, the high-sCD80 group had significant differences in 5MS and 5OS compared to the low-sCD80 group.

**Conclusions:**

In conclusion, sCD80 may negatively affect systemic immune circumstances, in STS, and may have potential as a therapeutic target.

## Background

Soft tissue sarcoma (STS) is a rare, heterogeneous group of tumors [1]. The incidence of STS is fewer than six per 100,000 cancer cases, which represents 1–2% cases of all cancer in adults [1]. Despite recent advances in the diagnosis and treatment of STS, patients who develop metastasis have mortality rates. Therefore, many studies have attempted to define different factors for predicting the prognosis of patients with STS. Older age and deep, truncal, high-grade, and large size of STS have been reported to be prognostic factors that are linked to poor prognosis [2–4]. In addition, the formation of an immune escape mechanism that suppresses the immune response to the tumor is known to have a significant effect on its prognosis. Immune checkpoint protein (ICP), which is a central factor group of the immune system, has been reported to have a correlation between the degree of its expression and the prognosis of patients with malignant tumors, and many inhibitors have appeared as therapeutic targets [5]. C. Perisano et al. evaluated the immunohistochemistry of PD1/PD-L1 expression in 60 adult patients affected by high-grade sarcomas. It showed positivity among the different subgroups of positive PD1 lymphocytes infiltration and PD-L1 expression in high-grade sarcomas [6].

On the other hand, a soluble form of ICP in circulating blood induced systemic immunosuppression [7]. An association between the soluble form of PD-L1 (sPD-L1) and cancer has been reported, such as renal cell carcinoma, hepatocellular carcinoma, esophageal cancer, lung cancer, gastric cancer, rectal cancer, and lymphoma [8–14]. Elevated sPD-L1 had significant relation with exacerbation of prognosis in cancer patients including STS [15]. In this study, we investigated the relationship between the soluble form of CD80 (sCD80) which is a ligand for the inhibitory system CTLA-4, in blood and clinicopathological parameters in patients with soft tissue tumors.

## Materials and methods

### Patients

A total of 119 patients who were treated were retrospectively reviewed, including 34 patients with benign soft tissue tumors and 85 patients with STS from 2002 to 2016, and were enrolled in this study. Patients who had local recurrence or who were referred for additional resection after inadequate resection in a previous hospital or who had distant metastasis at the first visit were excluded from this study. This study was approved by the Ethics Committee of the Mie University Graduate School of Medicine. All procedures performed in studies involving human participants were in accordance with the ethical standards of the Ethics Committee of Mie University and with the 1975 Declaration of Helsinki. The histopathological diagnosis and histological grade were verified by independent pathologists.

### sCD80 measurement

Blood samples from all patients were obtained prior to initial treatment. All samples were stored at -80 degrees until measurement followed by centrifugation at 1000 × *g* for 15 min. sCD80 levels were measured using Human CD80 ELISA Kit (B7-1) (Abcam, Cambridge, UK). The minimum detectable level of sCD80 was 31 pg/mL; values under the detectable level were assigned a value of 0 pg/mL.

### Statistical analysis

Statistical analysis was performed to compare the serum sCD80 levels to various clinical parameters using the Mann–Whitney U test or the Kruskal–Wallis test for quantitative data. To evaluate the threshold for detecting recurrence, metastasis, or death due to disease, receiver operating characteristic (ROC) curve analysis was performed. The ROC curves were created by plotting sensitivity on the y-axis and the false-positive rate (1-specificity) on the x-axis, and the area under the curve (AUC) was assessed. Local recurrence-free survival (LRFS) was defined as the time from the initial treatment to the date of clinically documented local recurrence. Metastasis-free survival (MFS) was defined as the time from the initial treatment to the date of clinically documented distant metastasis. Overall survival (OS) was defined as the time from the initial treatment to the date of death attributed to the neoplasm. Kaplan–Meier survival plots and log-rank tests were used to assess the differences of LRFS, MFS, and OS. The correlation between immunostaining and sCD80 test results was evaluated by the kappa coefficient test. To adjust for the imbalance in prognostic factors among patients, Cox proportional hazard analysis was used. *p* < 0.05 was considered significant. The EZR software program was used for statistical analyses.

## Results

### Characteristics of the study population

The clinical and pathological characteristics of the study population are summarized in Table 1.

**Table 1:** Characteristics of patients with soft tissue tumors

Characteristics		Healthy volunteers (16)	Benign (34)	STS (85)	*p* value
Sex	Male	8	18	45	
	Female	8	16	40	
Age	Average (SD)	45.0(14.0)	54.3(13.3)	63.4(15.2)	#p < 0.001
sCD80	Average (SD)	510.5(128.1)	566.8(187.9)	609.7(499.0)	#0.368
Characteristics in benign and STS patients	N(119)	sCD80 average (SD)	p value		
Sex	Male	62	580.5(335.3)	**0.836	
	Female	57	616.0(521.5)		
Age	< 60y	53	540.6(473.1)	**0.0295	
	> 60y	66	643.2(395.6)		
History of other malignant tumors	-	95	587.1(450.6)		
	+	24	638.5(360.3)		

Sex, age, malignancy, and sCD80 values were evaluated by *Fisher’s exact test and the #Kruskal–Wallis test. sCD80 values were compared for each parameter by the **Mann–Whitney test

Age was significantly different between healthy volunteers, the patients with benign tumors, and the patients with STS. There was no significant difference in sCD80 levels between healthy volunteers, the patients with benign tumors, and the patients with STS. The histopathological diagnosis of the 34 benign tumors was 12 lipomas, 14 schwannomas, 3 myxomas, 2 tenosynovial giant cell tumors, 1 leiomyomas, and 1 others, while those of the 85 STSs were 38 liposarcomas (22 well-differentiated liposarcomas (WLSs), 12 dedifferentiated liposarcomas (DLSs), and 4 myxoid liposarcomas (MLSs)), 13 myxofibrosarcomas (MFSs), 12 undifferentiated pleomorphic sarcomas (UPSs), 8 leiomyosarcomas (LMSs), 5 synovial sarcomas (SSs), 4 malignant peripheral nerve sheath tumors (MPNSTs), and 5 others (Table 2). All patients with benign tumors underwent tumor resection, and 85 patients with STSs received treatment (wide resection in 57 patients, marginal resection in 23 patients, intralesional resection in 3 patients, and ion beam radiotherapy in 2 patients) (Table 3). Although male patients over 60 years old and those with a history of other malignant tumors had higher sCD80 levels, there was only a significant difference in sCD80 levels for age in benign and STS patients (Table 1).

**Table 2:** Histological classification of tumors

Histology	n
WLS	22
MFS	13
UPS	12
DLS	12
LMS	8
SS	5
MPNST	4
MLS	4
Others	5

**Table 3:** Characteristics of patients with STS. sCD80 values were compared for each parameter in STS patients

Characteristic in STS patients		N (85)	sCD80 median	*p *value
Sex	Male	45	495.4	*0.604
	Female	40	474.8	
Age	< 60y	29	320.9	*0.00244
	> 60y	56	526.9	
Tumor size	< 10 cm	42	486.5	*0.93
	> 10 cm	43	474.8	
Location	Extremity	61	452.3	*0.095
	Trunk	24	639	
Tumor depth	Superficial	13	531.1	*0.132
	Deep	72	469.6	
Histological grade	Low grade	26	431.2	*0.292
	High grade	59	496.4	
Stage	I	26	431.2	#0.541
	II	15	496.4	
	III	44	508.6	
Treatment	Wide resection	57	494.5	#0.253
	Marginal resection	23	381.9	
	Intralesional resection	3	592.1	
	Ion beam radiotherapy	2	382.1	
Chemotherapy	-	59	497.3	*0.198
	+	26	451.3	
Radiotherapy	-	64	463.5	*0.439
	+	21	562.1	
History of other malignant tumors	-	66	457.4	*0.056
	+	19	562.1	

^*^Mann–Whitney test, #Kruskal–Wallis test

### Characteristics of the STS population

**Fig. 1:**
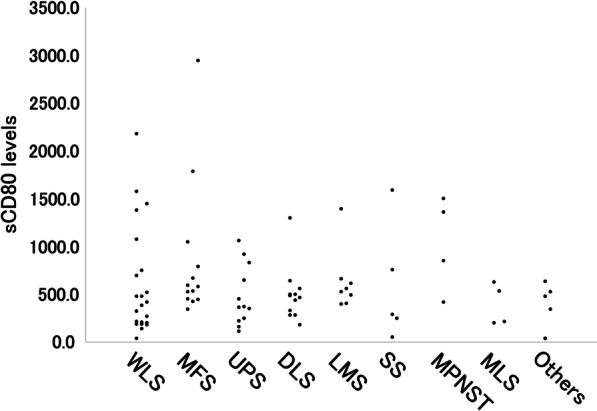
Scatter plot showing the sCD80 values for each histology

The clinical and pathological characteristics of the STS patients are shown in Table 3. There was a significant difference in sCD80 levels for only age. By histopathological subgroups, the average (standard deviation) of sCD80 levels was as follows: MPNST 1033.6 (495.5) pg/mL; MFS 856.2 (734.0) pg/mL; UPS 476.7 (314.0) pg/mL; SS 586.1 (616.9) pg/mL; WLS 604.5 (571.6) pg/mL; DLS 497.0 (284.7) pg/mL; MLS 394.2 (218.1) pg/mL; LMS 631.5 (320.5) pg/mL; and others 403.9 (230.8) pg/mL (Fig. 1). According to the AJCC classification of STSs, 26 patients were classified as stage I, 15 were classified as stage II, and 44 were classified as stage III. The average of sCD80 concentrations tended to be higher with higher stages than with lower stages, but the difference was not significant.

### Recurrence, metastasis, and death of disease in the STS group

The mean follow-up in malignant patients was 40 months (range 0.6–208 months). During the period of this study, 23 patients developed recurrence, 36 patients developed metastasis, and 25 patients died of disease.

**Fig. 2:**
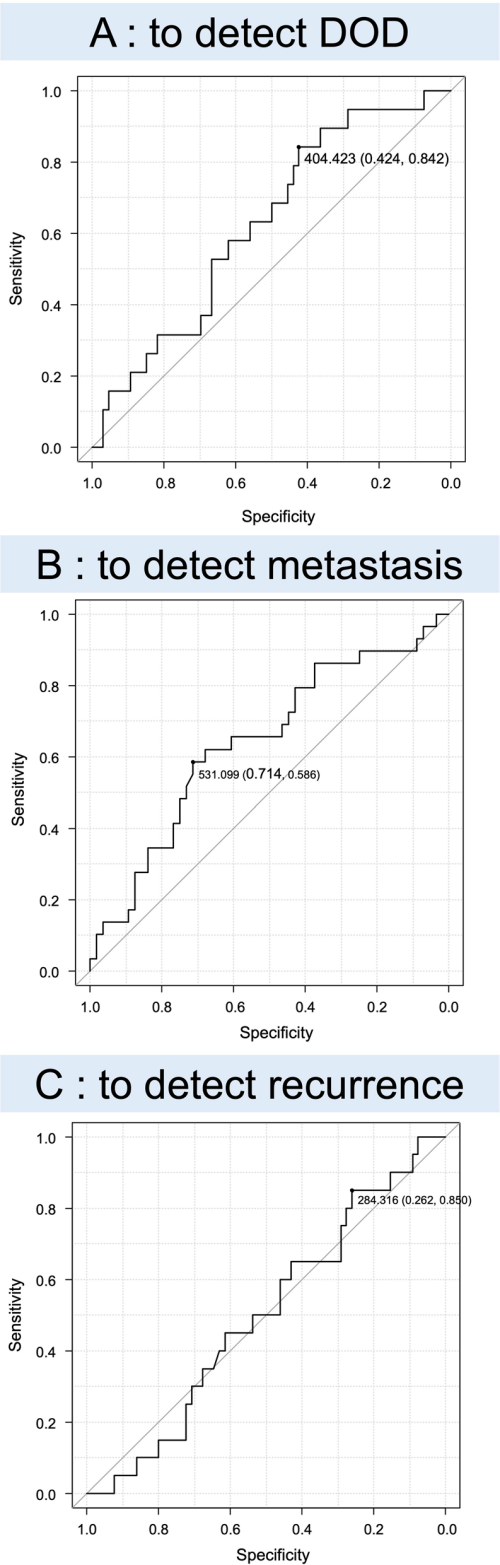
Receiver operating characteristic curve analysis. Diagnostic accuracy is evaluated by the area under the curve for identifying DOD (**A** AUC: 0.625, 95% CI 0.49–0.761), metastasis (**B** AUC: 0.641, 95% CI 0.514–0.767), and recurrence (**C** AUC: 0.50, 95% CI 0.362–0.639). A cutoff of 404 pg/mL results in sensitivity of 84.2% and specificity of 42.4% for identifying DOD, a cutoff of 531 sensitivity of 58.6% and specificity of 71.4% for identifying metastasis, and a cutoff of 284 sensitivity of 85.0% and specificity of 26.2% for identifying recurrence

**Fig. 3:**
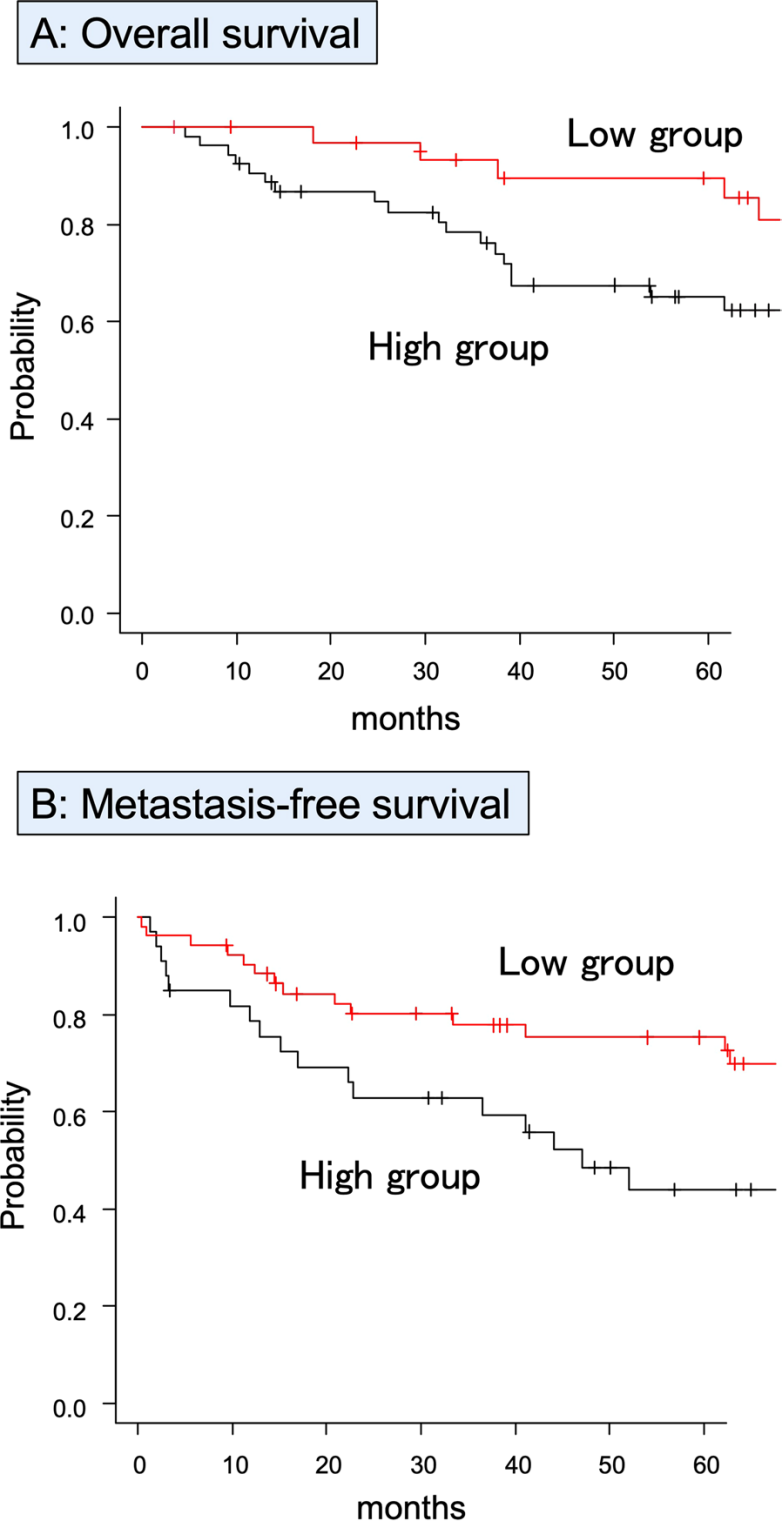
Kaplan–Meier analysis for STS. 5OS (**A**) and 5MFS (**B**) compared in the low- and high-sCD80 groups are shown by Kaplan–Meier analysis. The high-sCD80 group has significantly lower 5MFS (low sCD80 75.3%, high sCD80 44.0%, *p* = 0.016). For 5OS, the high-sCD80 group has a significantly worse prognosis (low sCD80 89.5%, high sCD80 65.0%, *p* = 0.015). The x-axis indicates months

To confirm the diagnostic accuracy of sCD80 for identifying metastasis, DOD, and recurrence, ROC analysis was performed by evaluating the area under the curve (AUC). The AUCs for identifying DOD, metastasis, and recurrence were 0.641 (95%CI 0.514–0.767), 0.625 (95%CI 0.49–0.761), and 0.50 (95%CI 0.362–0.639), respectively (Fig. 2A–C). Based on the ROC analysis, a cutoff value of 404 pg/mL was used to divide the groups into low (≤ 404 pg/mL) and high (> 404 pg/mL) sCD80 groups for 5OS. High group had a poorer 5OS than low group (Fig. 3A, low sCD80: 89.5%, high sCD80 65.0%, *p* = 0.015). Like 5OS, to divide sCD80 into two groups for 5MFS, a cutoff value of 531 pg/ml was used to divide the groups into low (≤ 531 pg/ml) and high (> 531 pg/ml) sCD80 groups. The high group had significantly lower 5MFS (Fig. 3B, low sCD80: 75.3%, high sCD80: 44.0%, *p* = 0.016).

In the same way, to divide sCD80 into two groups for 5LRFS, ROC analysis was performed. As AUC was 0.5 for detecting recurrence, further analysis was not evaluated.

### sCD80 for high-grade STS

**Fig. 4:**
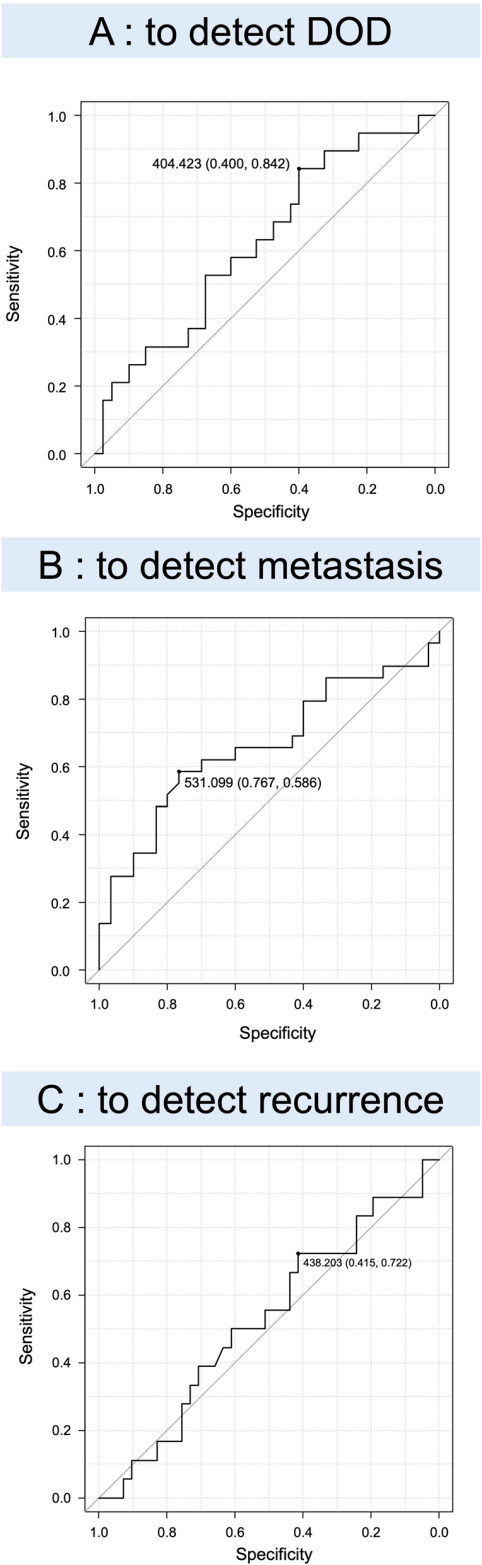
Receiver operating characteristic curve analysis. Diagnostic accuracy is evaluated by the area under the curve for identifying DOD (**A** AUC: 0.621, 95% CI 0.467–0.775), metastasis (**B** AUC: 0.661, 95% CI 0.518–0.805), and recurrence (**C** AUC: 0.525, 95% CI 0.364–0.686). Excluded the results of 5LRFS because the AUC of 5LRFS was low (AUC 0.525). A cutoff of 404 pg/mL results in sensitivity of 84.2% and specificity of 40.0% for identifying DOD and a cutoff of 531 sensitivity of 58.6% and specificity of 76.7% for identifying metastasis

**Fig. 5:**
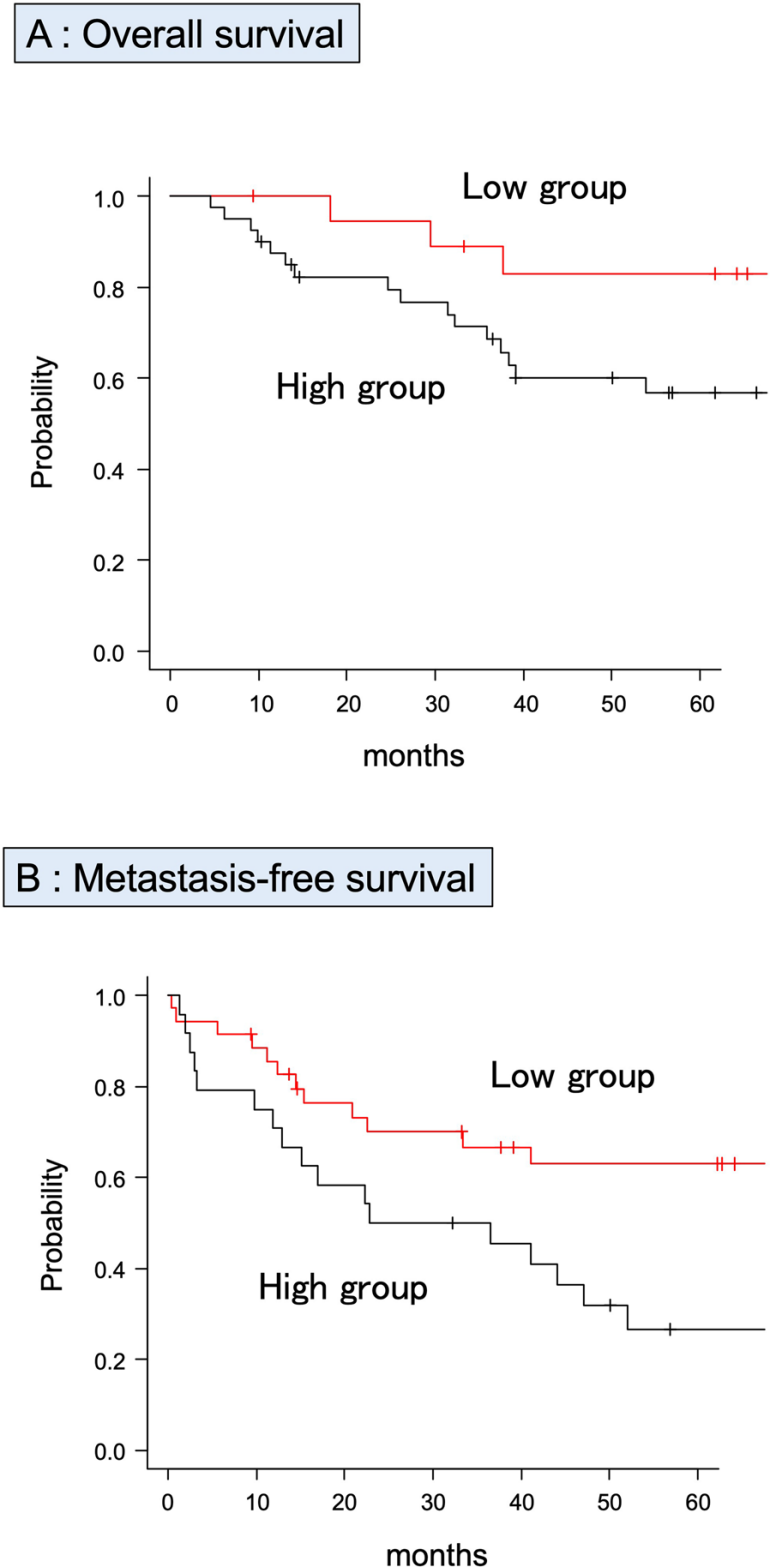
Kaplan–Meier analysis for STS. 5OS (**A**) and 5MFS (**B**) compared in the low- and high-sCD80 groups are shown by Kaplan–Meier analysis in patients with high-grade STS. 5OS shows no significant difference (5OS: low sCD80 83.0%, high sCD80 56.3%, *p* = 0.617). The high-sCD80 group has significantly lower 5MFS (low sCD80 62.9%, high sCD80 26.5%, *p* = 0.017). The x-axis indicates months

Next, we excluded 23 patients with well-differentiated liposarcoma from our cohort due to their excellent prognosis and examined the relationship between sCD80 value and survival in the remaining 59 patients. The median sCD80 value was 496.4 pg/mL (mean = 611.0, range = 48.8–2947.4) in 59 patients. During the period of this study, 20 patients developed recurrence, 36 patients developed metastasis, and 25 patients died of disease. Like above, to divide sCD80 into two groups for 5OS and 5MFS, a cutoff value of 404 and 531, respectively (Fig. 4A, B.), was used to divide the groups into low- and high-sCD80 groups (Fig. 5A, B). The results of 5LRFS were excluded because the AUC of 5LRFS was low (AUC 0.525) (Fig. 4C).

High group had tend to be a poorer 5OS than low group (low sCD80: 83.0%, high sCD80 56.3%, *p* = 0.062). The high group had significantly lower 5MFS (low sCD80: 62.9%, high sCD80: 26.5%, *p* = 0.022). (Fig. 5A, B). In multivariate Cox proportional hazard analysis, sCD80 showed significant difference for 5MFS (HR:2.397, 95% CI 1.143–5.027; *p* = 0.021) (Table 4).

**Table 4:** Prognostic factors for 5-year metastasis-free survival (5MFS) in 59 patients with high-grade soft tissue sarcoma

Characteristic in high-grade STS patients		N (59)	Log-rank test 5MFS (%)	p value	Multivariate COX HR (95%CI)	p value
Sex	Male	30	51.2	0.69		
	Female	29	42.9			
Age	< 60y	18	57.2	0.28		
	> 60y	41	43.2			
Tumor size	< 10 cm	32	51.2	0.49		
	> 10 cm	27	42.3			
Location	Extremity	48	50.3	0.21		
	Trunk	11	34.1			
Tumor depth	Superficial	7	42.9	0.93		
	Deep	52	48.1			
sCD80	< 531	35	62.9	0.017	1	0.021
	> 531	24	26.5		2.397 (1.143–5.027)	

## Discussion

Cancer cells, in order to avoid attacks from immune, aggressively use immunosuppressive function of regulatory T cells, bone marrow-derived suppressor cells, and the ICP.

Originally, ICP exists to suppress excessive activation of T cell and not to attack itself, but in the carcinogenic process, cancer cells are used to avoid attack from the immune system and proliferate. Currently, various immune checkpoint molecules and their ligands have been identified. After anti-CTLA4 antibody has been approved, it is becoming a new standard treatment for many cancer types such as melanoma and non-small cell lung cancer [16]. There are also some reports of clinical trials of checkpoint inhibitors in patients with STS and reports of phase II trials of ipilimumab, an anti-CTLA4 inhibitor, in patients with recurrent synovial sarcoma [17]. Despite high expression of CT (cancer–testis) antigens by synovial sarcomas of patients treated in the study, there was neither clinical benefit nor evidence of anti-CT antigen serological responses. In the other phase II trial, the response rate of the combination therapy of nivolumab and ipilimumab was higher (16%) than monotherapy of nivolumab (5%). This mean CTLA-4 is involved in tumor exacerbation of STS [18].

In this study, we focused on CD80, which is a suppressor ligand for CTLA4. CD80 is a transmembrane glycoprotein, a co-stimulator expressed on the surface of activated monocytes, B cells, and T cells [19, 20]. It has been reported that CD80 is also expressed in tumor cells and glomerular epithelial cells [21, 22].

The two B7 family molecules CD80 (B7-1) and CD86 (B7-2) are positioned as co-stimulatory molecules that play the most important role in T cell activation. CD80/86 activates T cells by binding to CD28, which is expressed on T cells; on the other hand, CTLA4 expressed on activated T cells and Treg cells binds to CD80/CD86 with a stronger affinity than CD28 and inhibits T cell activation. In addition, CTLA4 removes CD80/CD86 from APC (antigen-presenting cell), thereby inhibiting co-stimulation signals and suppressing T cell activation [19].

In these days, soluble form of ICPs attracts attention. High level of circulating soluble PD-L1 is significantly correlated with poor prognosis of many malignant neoplasm including STS [1, 9–15]. Because soluble PD-L1 functionally suppresses systemic immune response, therapeutic targets have been expanding from cellular PD-L1 to cellular and circulating soluble PD-L1 [23]. In this study, we analyzed the relation between soluble CD80 and prognosis in STS patients to present a hypothesis that soluble CD80 may affect for immune system in STS.

There are three reports of the origin of sCD80: shedding of CD80 on the cell membrane [24], CD80 on microvesicles released from cells [25], and splicing variants of CD80 [17]. Of the several splicing variants, the deleted form of the transmembrane domain is released unstable on the cell membrane, resulting in sCD80.

Functionally, the bifacial effect of sCD80 on tumor behavior has been reported. In one aspect, sCD80 inhibits T cell activation by preferentially binding to CTLA4 [19] and elevated levels of sCD80 have been a poor prognosis in patients with blood cancer and prostate cancer [25]. In another aspect, sCD80 inhibits the suppression of T cell activation [26], induces T cell proliferation [27], and inhibits PD-L1/PD1-mediated immunosuppression [28]. In this study, we successfully demonstrated that higher sCD80 was associated with shortened survivals and metastasis-free survival. sCD80 may affect immune system suppressively in STS. To date, seven clinical trials of galiximab, an anti-CD80 antibody, have been conducted (https://clinicaltrials.gov). In one of the lymphoma clinical trials, it has been reported that 49% of patients showed a decrease in tumor volume after administration of galiximab, and there were no grade 4 adverse events or treatment-related deaths [29]. In this study, the high sCD80 value had a poor prognosis, so it is possible that the effect of galiximab can be expected even in the STS region.

## Limitations

This study has some limitations. The number of patients was few. STS is rare cancer, a heterogeneous group of tumors. The incidence of STS is fewer than six per 100,000 cancer cases, which represents 1–2% cases of all cancer adults [30]. Therefore, it is difficult to collect samples and may be difficult to decide its cutoff value. In this study, the cutoff value was decided by the ROC curve. More samples may give us a more correct cutoff value of sCD80 and decide the cutoff value in STS. We believe that the level of sCD80 could be a useful prognostic marker in patients with STS.

## Data Availability

Not applicable.

## Abbreviations

ICP: Immune checkpoint protein
sCD80: Soluble form of CD80
STS: Soft tissue sarcoma
MS: Metastasis-free survival
OS: Overall survival
sPD-L1: Soluble form of PD-L1
ROC: Receiver operating characteristic
AUC: Area under the curve
LRFS: Local recurrence-free survival
WLS: Well-differentiated liposarcoma
DLS: Dedifferentiated liposarcoma
MLS: Myxoid liposarcoma
MFS: Myxofibrosarcoma
UPS: Undifferentiated pleomorphic sarcoma
LMSs: Leiomyosarcoma
SS: Synovial sarcoma
MPNST: Malignant peripheral nerve sheath tumor
DOD: Died of disease
